# Ectopic Opening of Common Bile Duct into Duodenal Bulb and Gastric Antrum and Its Importance

**DOI:** 10.34172/aim.2022.110

**Published:** 2022-10-01

**Authors:** Ümit Karabulut, Ali Üzel, Ramazan Yolaçan, Feyzullah Uçmak, Muhsin Kaya

**Affiliations:** ^1^Department of Gastroenterology, Dicle University, Faculty of Medicine, Diyarbakır, Turkey

**Keywords:** Ectopic opening, Cholangitis, Common bile duct

## Abstract

**Background:**

Ectopic opening of the common bile duct (CBD) is extremely rare, and its importance has not been adequately defined. The aim of our study is to present the characteristics of patients with this abnormality.

**Methods:**

This retrospective study was conducted in a tertiary center in Dicle University Hospital, Diyarbakır, Turkey, between October 2008 and December 2020. We present clinical, laboratory, endoscopic and cholangiographic features as well as the success rate of therapeutic interventions of consecutive patients with this abnormality undergoing endoscopic retrograde cholangiopancreatography (ERCP).

**Results:**

Ectopic opening of the CBD was identified in 29 (21 men; mean age 62) out of 3872 (0.74%) patients. There was a history of cholecystectomy in 14 (48%) and recurrent acute cholangitis in 20 (69%) patients. We found peptic ulcer in 13 (45%) and duodenal deformity associated with apical stenosis in 21 (72%) patients. Opening site was seen as abnormal in all patients, and it opened into the antrum in 2 (6.8%) and into the first part of the duodenum in 27 (93%) patients. Copious amount of bile and/ or bile sediment in the stomach were seen in all patients. We observed dilatation in both intrahepatic and extrahepatic bile ducts together with tapered narrowing and a hook-shaped distal end of CBD in all patients. There was bile stone in 26 (89%) and sludge in 3 (10%) patients. Sphincterotomy was not performed in any patients because there was not enough incision distance. Balloon dilatation was performed for extraction of stone and sludge in all patients. Complete stone extraction was achieved in only 7 out of 26 (27%) patients.

**Conclusion:**

Ectopic opening of CBD is usually associated with gastroduodenal and bile ducts disease. Endoscopic treatment is unsatisfactory in most patients with this abnormality.

## Introduction

 The common bile duct (CBD) normally enters the second part of the duodenum and opens into the ampulla of Vater. Ectopic openings of CBD into the antrum and into the first part of the duodenum have been increasingly detected after widespread use of endoscopic retrograde cholangiopancreatography (ERCP). Ectopic opening of the CBD into the first part of the duodenum, pyloric canal and antrum have been reported previously. Its clinical features as well as endoscopic and cholangiographic findings have been reported.^1-13^ Increased incidence of duodenal ulcer, stenosis in the duodenal bulbus, dilatation in the biliary tract and bile stone formation have been reported.^1,3,14,15^ Most patients with this abnormality have clinical signs and symptoms related to acute cholangitis and biliary obstruction caused by bile stone and sludge. Some patients may have signs and symptoms of duodenal ulcer and gastric outlet obstruction.^1,3,15^ Most of the literature related to this abnormality are published from Far East countries. Failure of the procedure because of duodenal stenosis and inability to detect papilla at its classical location may explain why this abnormality is rarely reported. The aim of our study was to comprehensively define the clinical, endoscopic, and cholangiographic features of our case series.

## Material and Methods

 We collected the endoscopic and cholangiographic findings of patients who underwent ERCP in our hospital between October 2008 and December 2020. The demographic, clinical, laboratory, endoscopic and cholangiographic findings of all patients with ectopic opening of CBD were recorded. We also collected all data about therapeutic interventions. All patients were followed up regularly every 3 to 6 months. If patients had more than one admission to our department, all data related to the first admission were presented. Endoscopic and cholangiographic findings were recorded prospectively. Ectopic opening of CBD into the first part of the duodenum, stomach and third part of the duodenum was defined as (1) inability to see the ampulla of Vater in its normal location in the second part of the duodenum; (2) demonstration of ectopic opening of CBD in the first part of the duodenum or into the stomach by both duodenoscopy and cholangiography; (3) no evidence of another connection between bile ducts and the duodenum. A gastroscope was used instead of duodenoscope in patients with duodenal stenosis that was too advanced to allow passage of the duodenoscope. Then, the presence of ectopic opening abnormaly was confirmed by examining the entire duodenum with the gastroscope. In patients with ectopic opening, endoscopic sphincterotomy was not performed because there was not enough incision distance around the orifice. Therefore, the orifice of CBD was dilated by a pyloric dilatation balloon (12–18 mm in diameter) before therapeutic intervention in all patients. A nasobiliary drain or plastic stent was placed to prevent the development of acute cholangitis in patients whose gallstones could not be removed.

## Results

###  Demographic and Clinical Features

Table 1 shows the demographic, clinical and laboratory features of patients. The ectopic opening abnormaly of the CBD was identified in 29 (21 men, 8 women; mean age 62; range 17–86 years) out of 3872 (0.74%) patients who underwent ERCP during the 12-year period. There was a history of cholecystectomy in 14 out of 29 (48%) and recurrent acute cholangitis in 20 (69%) patients. The most common symptoms were recurrent epigastric and right upper quadrant pain, nausea, vomiting, fever and chills that are compatible with acute cholangitis. Two patients (6.8%) also had acute cholecystitis and one patient also had ruptured *Echinococcus granulosus* during the first admission. There was a history of duodenal peptic ulcer in 11 (37%) patients. One patient (man, 63 years old) with a history of cholecystectomy 20 years ago, had compensated secondary biliary cirrhosis associated with esophageal varices and portal hypertensive gastroduodenopathy.

**Table 1:** Demographic, Clinical and Laboratory Characteristics of Patients During the First Admission

**Characteristics**	
Age (mean; range)	62 (17–86)
Men/Female	21/8 (72%/28%)
Parameter, No. (%)	
Cholecystectomy	14 (48)
History of recurrent cholangitis	20 (69)
History of peptic ulcer	13 (44)
Symptoms, No. (%)	
Abdominal pain	29 (100)
Fever	20(68)
Icterus	20 (68)
Nausea	11 (37)
Vomiting	11 (37)
Laboratory parameters, No. (%)	
Leukocytosis	13 (44)
Anemia	8 (27.5)
Elevated ALT	24 (82)
Elevated AST	23 (79)
Elevated ALP	16 (55)
Elevated GGT	25 (86)
Elevated total bilirubin	22 (75)

ALT, alanine aminotransferase; AST, aspartate aminotransferase; ALP, alkaline phosphatase; GGT, gamma glutamyl transferase.

###  Laboratory Findings

 We found elevated white blood cells (range 4.82–35.7×10^9^/L; normal values 4.5–10×10^9^/L) in 13 (44%) patients, decreased hemoglobin level (range 8.07–15.2 g/dL, normal values 12–14 g/dL) in 8 (27.5%), elevated aspartate aminotransferase (range 19–484 U/L; normal values, 10–35 U/L)in 23 (79%), alanine aminotransferase (range 13–533 U/L; normal values, 10–40 U/L) in 24 (82%), alkaline phosphatase (range 52–1237 U/L; normal values, 40–150 U/L) in 16(55%), gamma glutamyl transferase (range 17–1483 U/L; normal values, 9–64 U/L) in 25(86%) and total bilirubin level (range 0,26–17 mg/dL; normal values, <1,2 mg/dL) in 22(75%) patients.

###  Endoscopic Findings 

**Figure 1:**
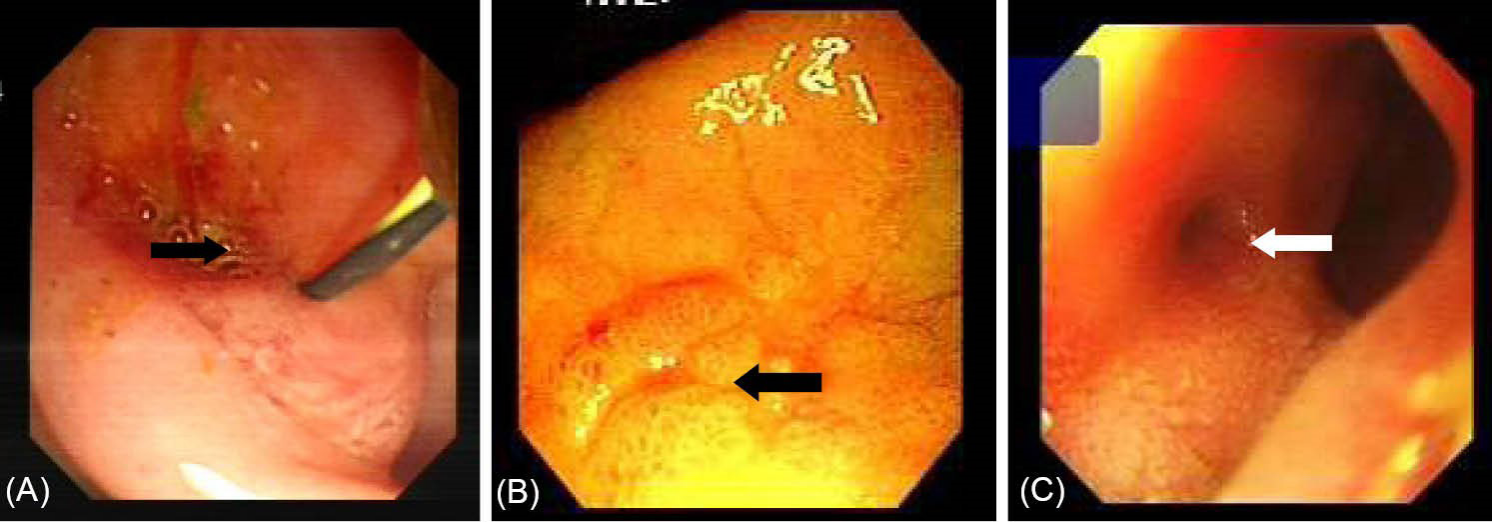
** (A) **Opening site of CBD shows a slit-like opening into the duodenal bulb. **(B) **Opening site of CBD shows an ill-defined mucosal edematous area in the duodenal bulb (black arrow). **(C)** Endoscopy shows the circular hole-like appearance of ectopic opening of CBD into the duodenal bulb (white arrow).

**Figure 2:**
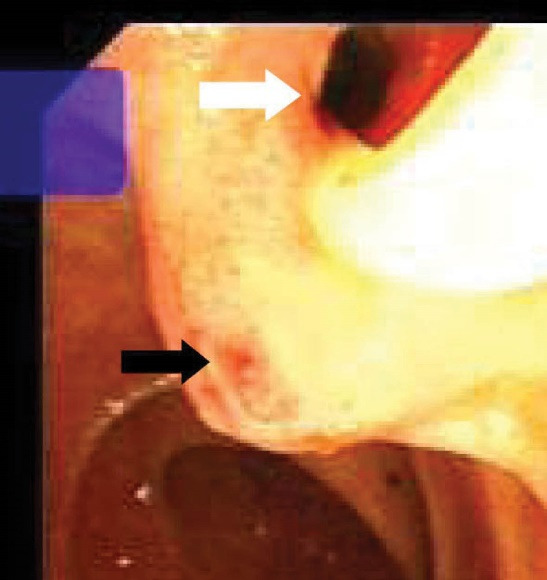
Endoscopy shows the slit-like appearance of ectopic opening of CBD (white arrow) and nipple-like appearance beneath it (black arrow) into the duodenal bulb.

**Figure 3:**
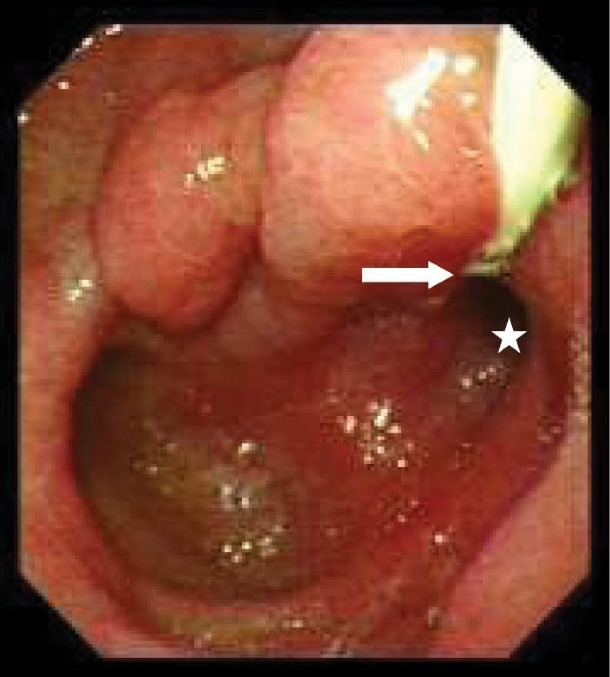
Endoscopy shows saccular dilatation and deformity of the duodenal bulb associated with apical stenosis (asterisk). Ectopic opening of CBD located to the beginning of stenosis (arrow).

Table 2 shows detailed information about the endoscopic and cholangiographic findings and therapeutic procedure in all patients. Peptic ulcer in different stages, ranging from a scar mark alone to a crater with varying size, was found in the duodenum in 11 (37%) patients. In two patients (6.8%), we observed gastric peptic ulcer (3×2 cm and 2×1 cm in diameter, respectively) located to the incisura angularis and its benign nature was confirmed by biopsy. Ectopic opening opened into the antrum in 2 (6.8%) patients and into the first part of the duodenum in 27 (93%) patients. Both patients with ectopic opening into the first part of the duodenum and into the antrum did not have the classic appearance of the ampulla of Vater and there was a slit-like appearance (Figure 1A), ill-defined mucosal edematous area (Figure 1B) or a circular hole (Figure 1C). In patients with ectopic opening located in the first part of the duodenum, the orifice was located in the posterior wall of the first part of the duodenum. In two patients with ectopic opening located in the antrum, the orifice was located between the incisura angularis and pyloric canal. In 5 out of 29 (17%) patients, the bile duct and pancreatic duct opened to the duodenal bulb separately (Figure 2). All patients had abnormal appearance of the duodenal bulb. Although there was no duodenal ulcer in those patients with ectopic opening to the antrum, both patients had duodenal deformity and apical stenosis. Duodenal deformity associated with apical stenosis was found in 21 out of 29 (72%) patients (Figure 3). None of patients with apical stenosis required balloon dilation. Eight out of 21 (38%) patients with apical stenosis had active duodenal ulcer. Thirteen out of 21 (62%) patients with duodenal deformity and apical stenosis had no visible active ulcer or scar in their duodenum and antrum. Copious amount of bile and/or bile sediment in the stomach was seen in all patients.

**Table 2:** Endoscopic and cholangiographic findings of patients during the first admission

**Parameter**	**Number of Patients (%)**
Endoscopic findings	
Duodenal ulcer	11 (37)
Apical stenosis	21 (72)
Gastric ulcer	2 (6.8)
Abnormal appearance of opening site	29 (100)
Excessive bile in gastric lumen	29 (100)
Common bile duct opening into the duodenal bulb	27 (93)
Common bile duct opening into the antrum	2 (6.8)
Cholangiographic findings and therapeutic intervention	
Dilatation of intrahepatic bile ducts	29 (100)
Dilatation of extrahepatic bile ducts	29 (100)
Balloon dilatation of orifice	29 (100)
Bile stone	26 (89.6)
Complete stone extraction	7 out of 26 (26.9)
Stent placement	7 (24)
Nasobiliary drain placement	12 (41)

###  ERCP Findings and Therapeutic Intervention

**Figure 4:**
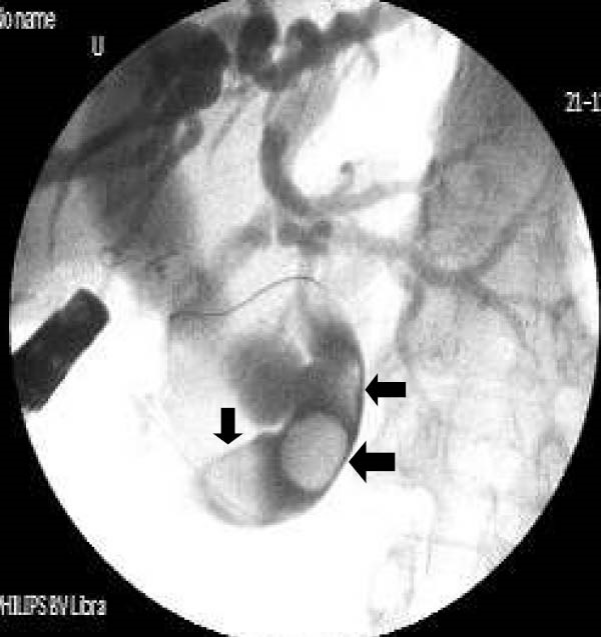
Cholangiography shows multiple stones (black arrows) and narrowing, a hook-shaped appearance in the distal end of CBD associated with elongated, dilated and folded extrahepatic and dilated intrahepatic bile ducts.

**Figure 5:**
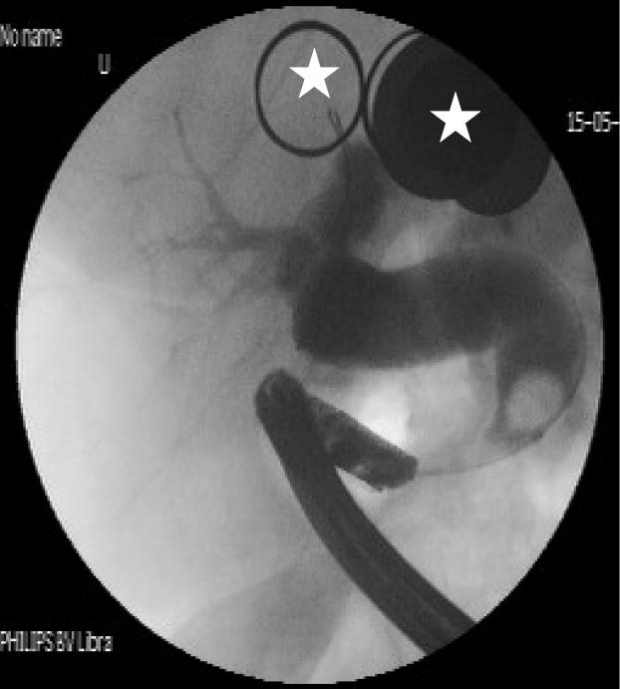
Cholangiography shows tapered narrowing, a hook-shaped appearance and bile stone in the distal end of CBD associated with intrahepatic and extrahepatic biliary dilatation (white asterisk show metal on patient’s dress).

Table 2 shows detailed cholangiographic information about all patients. The indication of ERCP was recurrent acute cholangitis associated with bile duct dilatation in 20 patients and abdominal pain associated with elevated liver enzymes and dilatation of bile ducts in 9 patients. CBD cannulation was successful in all patients and we did not use needle knife papillotomy for cannulation in any patients. We could not pass the duodenoscope to the second portion of the duodenum because of apical stenosis in 5 patients; we used a gastroscope (because it is thinner than a duodenoscope) to visualize the second and third portion of the duodenum in those patients and confirmed that the opening site of CBD was located above the apical stenosis site. CBD cannulation and therapeutic intervention were performed with the duodenoscope in all patients. Cholangiography showed diffuse dilatation of the extrahepatic bile duct (median 17 mm: range 12 to 40 mm in diameter,) and intrahepatic bile ducts in all patients. The dilated CBD showed tapered narrowing and a hook-shaped distal end in all patients (Figure 4). Twenty-six out of 29 (89%) patients had bile stone (Figure 5) and three (10%) patients had sludge in bile ducts. We did not perform sphincterotomy in any patients because there was not enough incision distance. After cholangiography, we dilated the ectopic opening orifice with a pyloric dilatation balloon (range 12–18 mm in diameter) in all patients for stone and sludge extraction and for free biliary drainage. Complete stone extraction was achieved in 7 out of 26 (27%) patients with bile duct stone. In 19 patients whose stone could not be retrieved because of acute angulation, tapered narrowing and a hook-shaped distal end of CBD and un-effective pull back of balloon catheter for extraction of stones, a nasobiliary drain (n = 12 patients) or stent (n = 7) was placed for prevention of cholangitis. We did not observe any complications such as bleeding, perforation and acute pancreatitis related to ERCP and balloon dilatation. The pancreatic ducts were opacified in 5 out of 29(17%) patients via separate opening site and there was not any communication between the pancreatic canal and bile ducts. We did not opacify the pancreatic canal in the remaining 24 patients because our primary aim was to perform biliary therapeutic intervention.

###  Clinical Follow-up

 During a mean follow-up of 27.1 ± 8.2 (range 3–54) months, all patients had at least one episode of acute cholangitis [mean 3.1 (range 1–5) episode]. Patients were admitted to the clinic and treated with intravenous fluid and antibiotics during acute cholangitis and then underwent ERCP for therapeutic intervention. Nine out of 19 patients whose stone could not be extracted, were operated during follow-up.

## Discussion

 Ectopic opening of the CBD into the first part of the duodenum was first reported by Fallopious in 1606 and a few case series have been published.^1-3^ Lindner et al, in their review of 1000 cholangiograms, did not report ectopic opening of CBD in the first part of the duodenum.^7^ Most of the literature on this abnormaly is from Far East and Turkey.^1-7,9,11,15^ This entity is not well-recognized in the world because some endoscopists might think that this abnormality is spontaneous bilioenteric fistula secondary to complications of peptic ulcer. Another reason why this abnormality is underreported may be related to the inability to perform ERCP because of inability to pass to the second part of the duodenum due to apical stenosis and/or because the ampulla of Vater is not in its normal localization in these patients. The true incidence of this anomaly is unknown, but has been reported in 8 of 5180 (1.5%),^2^ in 18 of 16541 (1%),^3^ in 53 of 12.158 (0.4%)^1^ and in 4 of 1040 (3.8%)^4^ patients who underwent ERCP. In our series, we identified ectopic CBD opening in 29 out of 3872 (0.74 %) patients who underwent ERCP during a 12-year period.

 The cause of ectopic opening of CBD is not known. It has been suggested that ectopic bile duct opening develops because of abnormal organogenesis.^3^ Most of the patients with this abnormality have chronic duodenal peptic ulcer, duodenal deformity and apical stenosis.^2^ One plausible mechanism for development of this abnormality is that its development may be related to increased fibrosis formation secondary to chronic duodenal ulcer and deformed bulb may retract the major papilla toward the duodenal bulb. However, the absence of the typical configuration of a papilla of Vater, the presence of a slit-like orifice of CBC and the absence of peptic ulcer in some patients with this abnormality support the possibility that this abnormality may be a congenital developmental abnormality rather than an acquired abnormality.

 This opening anomaly is diagnosed by visualization of the opening site of CBD by endoscopy and cholangiography. The typical appearance of the ampulla of Vater is absent and a slit-like orifice is seen during endoscopic examination. This slit-like opening configuration may reflect a poorly developed or absence of the sphincter of Oddi. In a majority of patients with ectopic opening of CBD into the first part of the duodenum, there is apical stenosis associated with duodenal ulcer.^1,11,14-17^ There are a few case reports showing that an opening of the CBD into the antrum might cause gastric ulcer.^5,10^ Excessive bile, bile precipitates^1,3^ and small gallstones^4,5^ in the stomach were detected in patients with ectopic opening of the CBD into the first part of the duodenum and into the antrum. The cause of apical stenosis and increased incidence of peptic ulcer in patients with this abnormality is unknown. It has been speculated that bile acids usually precipitate at acidic pH and becomes harmless. However, under alkaline condition where the pH is higher, constant exposure of the duodenal bulb and gastric mucosa to bile acids can induce mucosal inflammation, ulcer and stenosis.^3^ In our case series, there were copious amounts of bile and/or bile sediment in the stomach and slit-like orifice in all patients. The presence of duodenal deformity together with apical stenosis in 21 out of 29 (72%) patients and active duodenal ulcer in 8 out of 21 (38%) patients with apical stenosis was compatible with previously reported findings in patients with this anomaly. Thirteen out of 21 (62%) patients with duodenal deformity and apical stenosis had no visible active ulcer or scar in their duodenum and antrum. These findings may support the possibility that the presence of apical stenosis in these patients may be related to a congenital abnormality or may be related to fibrosis caused by a completely healed duodenal ulcer.

 Patients with ectopic opening of the CBD usually have clinical symptoms and signs of biliary tract diseases. Dilatation of intrahepatic and extrahepatic bile ducts, a hook-shaped configuration of the distal end of the CBD and biliary stones were the most common ERCP findings in this anomaly. The most common clinical presentations in these patients are bile duct stones, recurrent cholangitis, obstructive jaundice and abnormal liver function tests.^1-6^ Normal sphincteric musculature is absent around the entrance of accessory bile ducts into the gastrointestinal tract.^14,18^ In our case series, we found diffuse dilatation in both extrahepatic and intrahepatic bile ducts in all patients. We did not identify any stricture preventing biliary drainage on cholangiography in any patients. Because of the presence of diffuse dilatation in bile ducts, we can speculate that in addition to acute angulation of the distal end of the CBD, sphincter dysfunction may impede normal bile stream and cause chronic bile stasis, bile duct dilatation and formation of biliary sludge and bile stone. Manometric measurement may yield more accurate information about sphincter functions in these patients.

 Bilioenteric fistula and surgical bilioenteric diversion should be considered in the differential diagnosis of ectopic opening of CBD.^2^ Detection of the ampulla of Vater and CBD in normal localization and configuration on ERCP is an important finding for this distinction.^4^ In all our patients, we confirmed the diagnosis of ectopic opening of CBD by direct visualization of bile flow from orifice, the absence of the ampulla of Vater in its original location and no evidence of another connection between bile ducts and the gastrointestinal tract.

 In patients with ectopic opening of CBD together with bile duct stones, it is usually difficult to remove the stones by ERCP. Normal sphincteric structure is absent around the opening site. Therefore, there is a high risk of perforation and bleeding during endoscopic sphincterotomy. For this reason, endoscopic sphincterotomy should be avoided in these patients. Endoscopic balloon dilatation may be an alternative to sphincterotomy in these patients.^1,11,12,15^ We showed that balloon dilatation is an effective and reliable intervention in patients with this abnormality. However, acute angulation, tapered narrowing and a hook-shaped distal end of CBD and deformity of the duodenal bulb usually prevent effective pull back of balloon catheter for extraction of stones. Therefore, we did not achieve complete stone extraction in most of our patients. Because most of the patients with this abnormality have recurrent cholangitis, large bile stones and apical stenosis, surgery may be an effective alternative treatment modality for patients whose stone could not be retrieved by endoscopic intervention.

 The important limitation of this study is that this is a single-center, observational, retrospective and relatively small case-series. Nevertheless, this study may update information related to the clinical, endoscopic, cholangiographic features as well as treatment modalities of this relatively rare abnormality.

 In conclusion, the possibility of ectopic opening of CBD should be considered when the ERCPist cannot see the ampulla of Vater in its normal location together with apical stenosis. Although apical stenosis may render the procedure difficult in this patient population, ERCP can be performed safely with balloon dilatation. Tapered narrowing and a hook-shaped configuration of the distal end of CBD are the main causes of failure to retrieve stones from bile ducts in these patients. The long-term effects of endoscopic and surgical therapeutic intervention should be compared in larger studies.
